# Clinical and histopathological studies on neurodegeneration and dysautonomia in buffalo calves during foot-and-mouth disease outbreaks in Egypt

**DOI:** 10.14202/vetworld.2021.1622-1630

**Published:** 2021-06-23

**Authors:** Yasmin Bayoumi, Nader Sobhy, Abdelkarem Morsi, Wafaa El-Neshwey, Nora El-Seddawy, Abdelmonem Abdallah

**Affiliations:** 1 Department of Animal Medicine, Faculty of Veterinary Medicine, Zagazig University, Zagazig, Egypt; 2 Department of Pathology, Faculty of Veterinary Medicine, Zagazig University, Zagazig, Egypt

**Keywords:** dysautonomia, foot-and-mouth disease virus, heat intolerance, hirsutism, malignant

## Abstract

**Background and Aim:**

Signs of dysautonomia were frequently observed in calves that died during foot-and-mouth disease (FMD) virus (FMDV) outbreaks in Egypt from 2015 to 2018. This study aimed to describe the clinical and histopathological features of the central nervous system in malignant cases of FMD and excluding possible concurrent bacterial, and bovine herpes virus 4 (BHV4) infections or both.

**Materials and Methods:**

In this study, 335 FMDV-infected buffalo calves aged 1-22 months were clinically examined and followed until recovery or death. Of the 335 calves, 134 died (malignant group) and 201 recovered after exhibiting classic symptoms of FMD (recover group). The calves were subjected to clinical examination. For the malignant group, several laboratory trials were conducted to assess the possible cause/s of dysautonomia-related viral, bacterial, or concurrent infections. Koch’s postulates and polymerase chain reaction were employed. Postmortem and histopathological examinations of nervous tissue were performed.

**Results:**

In the malignant group, signs of dysautonomia were observed before death, including partial or complete gut dysfunction, loss of anal sphincter tone, rapid breathing sounds, fluctuating body temperature, and cardiac arrhythmias. In the malignant group, histopathological examination of the spinal cord, pons, medulla oblongata, hypothalamus, cerebellum, and cerebrum revealed demyelination, neuronal degeneration, and focal areas of malacia and gliosis. The nervous tissue and heart samples from malignant cases were positive for serotype O FMDV.

**Conclusion:**

Findings revealed in this study support the existence of neurodegeneration induced by FMDV infection in buffalo calves.

## Introduction

The foot-and-mouth disease (FMD) is a highly contagious acute vesicular disease of cloven-hoofed animals caused by the FMD virus (FMDV). The FMDV is a member of the Genus *Aphthovirus* in the family Picornaviridae and consists of small, non-enveloped, and single-stranded RNA [1]. The highly contagious nature of the FMD and its significant economic impact due to the associated mortalities make it the world’s worst farm animal disease [2]. Death occurs from acute myocarditis and myocardial degeneration [3]. *Picornaviruses* include many neurotropic viruses, such as *Poliovirus*, non-polio *Enteroviruses* in humans, *Theiler’s murine encephalomyelitis* virus, and *encephalomyocarditis* virus. Infections caused by neurotropic viruses can irreversibly disrupt the structural and functional architecture of the central nervous system (CNS), leading to a poor or fatal prognosis [4,5].

Neurodegeneration is a pathological condition that may lead to the loss of structure or function of neurons, with various clinical and pathological expressions [6]. The affection of the brain may be accompanied by neurological or non-neurological signs since the hypothalamus is a region of the CNS that most closely communicates with virtually all physiological systems. This is obvious since the dysfunction of the hypothalamus is frequently associated with non-neurological symptoms similar to manifestations of endocrine, gastrointestinal, and gynecological disorders [7].

The autonomic nervous system (ANS) is connected to every organ in the body and controls many functions to maintain homeostasis. The ANS is fundamentally controlled by centers located in the hypothalamus, brain stem, and spinal cord and is responsible for controlling gastrointestinal motility, defecation, urination, sweating, body temperature, and many other body activities [8].

Dysautonomia is a full-body condition, exhibiting itself in many ways that indicate CNS involvement. In addition, viral infections leading to brain dysfunction are more prevalent than currently appreciated [9], and neurodegenerative diseases are not well understood in veterinary practice.

This study aimed to describe the clinical and histopathological features of the CNS in malignant cases of FMD and excluding possible concurrent bacterial, and bovine herpes virus 4 (BHV4) infections or both.

## Materials and Methods

### Ethical approval

This study was approved by the Research Ethics Committee of the NHTMRI (IRB No.:29-2017).

### Study period and location

This study was conducted during three successive FMDV outbreaks (2015-2016, 2016-2017 and 2017-2018 outbreaks), on six FMD infected farms in Dakahlia and Sharkia Provinces, Egypt. Samples were processed at the Department of Animal Medicine, Faculty of Veterinary Medicine, Zagazig university, Egypt.

### Animals

In this study, 335 FMDV-infected buffalo calves aged 1-22 months from three successive FMDV outbreaks were included in the study (Table-1). All animals were healthy before the FMDV outbreaks. The calves belonged to six FMDV-infected farms in the Dakahlia and Sharkia Provinces, Egypt. The animals were observed and clinically monitored throughout the clinical course. Daily observations were performed from the onset of symptoms until recovery, death, or decision to slaughter.

**Table-1:** Calves incorporated in the study: Location, number, age, and clinical outcome of the disease.

Outbreak date	Province	No.	Age (month)		Clinical outcomes		
	
			<6 M	>6 M (8–22)*	Death	Slaughter	Recovery
Dec. 2015 - Feb. 2016	Dakahlia	83	17	66	7	13	63
	Sharkia	50	20	30	15	8	27
Dec. 2016 - Feb. 2017	Dakahlia	101	28	73	13	30	58
	Sharkia	32	21	11	10	21	1
Nov. 2017- Mar. 2018	Dakahlia	54	10	44	9	3	42
	Sharkia	15	5	10	-	5	10
Total		335	101	234	54	80	201

* All recovery cases were aged 11-22 months

### Clinical examination

Preliminary general examinations were performed on appearance, behavior, mental state, posture, gait, muscle fasciculation, and detectable changes in animal hair [10]. Vital signs were monitored: Body temperature (rectal and axial temperatures), pulse rate, and respiratory rate were measured. Appetite, rumination, defecation, and urination were observed. On physical examination, the heart and lungs were auscultated. Examinations of the buccal cavity and claws were also performed. Rectal palpation was performed using either a lubricated index finger (digital) or a lubricated hand in a conical position according to the age of the animal.

### Pathological studies

Postmortem examinations were performed for all malignant cases immediately after death or slaughter. Tissue specimens (heart, lung, brain, or spinal cord samples) were obtained and fixed in 10% buffered formalin, after which histopathological slides were prepared and reviewed [11].

### Bacterial investigation

The general primer set 27F/1492R for the quantification of bacterial 16S rRNA gene with a 1.5-kb expected product size was used to look for bacterial infections (Table-2) [12-15]. Polymerase chain reaction (PCR) was performed in a 25-mL reaction mixture in an Eppendorf Thermocycler. The reaction condition was as follows: Initial denaturation at 95°C for 10 min, followed by 35 cycles of denaturation at 95°C for 1 min, annealing at 52°C for 1 min, and elongation at 72°C for 1 min. The reaction was terminated by a final elongation step at 72°C for 10 min.

**Table-2:** Primers sets used in PCRs.

Primer	Sequence (5’ to 3’)	Size (bp)	Reference
Universal 16S rRNA 27F 1492R	5′-AGAGTTTGATCCTGGCTCAG-3′ 5′-CTACGGCTACCTTGTTACGA -3′	1500	[12]
Foot-and-mouth disease virus RdRp-F RdRp-R	5′-TTCGAGAACGGCACDGTCGGA-3′ 5′-CACGGAGATCAACTTCTCCTG-3′	881	[13]
A-1C612F O-1C 124F R2B58 (A, O)	5′-TAGCGCCGGCAAAGACTTTGA-3′ 5′-ACCAACCTCCTTGATGTGGCT-3′ 5′-GACATGTCCTCCTGCATCTG-3′	815 1300	[14]
SAT-2F SAT-2R	5′-ACGGTGGGAAYGTTCAAGAG-3′ 5′-TTCAAGACCGGTGTCAGC-3′	931	[13]
BHV-4 Outer primer BHV4-F BHV4-R	5′-GTTGGGCGTCCTGTATGGTAGC--3′ 5′-ATGTATGCCCAAAACTTATAATATGACCAG-3′	567	[15]
Inner primer BHV4-F BHV4-R	5′-TTGATAGTGCGTTGTTGGGATGTGG-3′ 5′-CACTGCCCGGTGGGAAATAGCA-3′	260	

### Samples for molecular confirmation of FMDV

Three hundred and seventy-two saliva and mouth lesion swabs from each suspected infected calf in two provinces were collected and molecularly examined for FMDV infection. The lung, heart, brain, and spinal cord samples were collected from the 134 malignant cases after death or slaughter. Preventing contamination of the samples with blood was provided special attention, and brain samples were collected away from congested blood vessels. The samples were collected on 2-mL phosphate-buffered saline (PBS). The saliva and mouth lesion swabs were squeezed in PBS and then centrifuged to obtain supernatants, and the tissues were homogenized, followed by centrifugation to obtain supernatants. The RNA from these supernatants was extracted using the QIAamp Viral RNA Mini Kit (Qiagen, Germany).

### Reverse transcription (RT)-PCR for detecting FMDV

Extracted RNA was subjected to RT-PCR using the one-step RT-PCR Kit. Primer sets were selected for detecting 3D common genes of the FMDV with further identification by detecting 1D genes for every three endemic serotypes in Egypt (i.e., A, O, and SAT2) (Table-2).

### Nested PCR for detecting BHV4

Nested PCR was performed using primers to amplify the thymidine kinase gene of BHV4 from brain tissue as outlined in Table-2. The first reaction performed to amplify 567-bp amplicon was used as a template to amplify 260-bp amplicon in the second reaction. The initial denaturation was 95°C for 15 min, followed by 1 min of denaturation at 95°C, annealing at 55°C, and elongation at 72°C, followed by a final elongation step at 72°C for 10 min. 1 mL of the first PCR product was used as a template in the second PCR in 25-mL reaction mixture following the same procedures, except for the annealing step, which was adjusted to 58°C for 1 min.

### Koch’s postulates

To achieve modified Koch’s postulates for viruses [16], putative FMDV was isolated in pure culture, followed by mice inoculation and then re-isolation from these mice. The prepared supernatants from suspected samples were inoculated into baby hamster kidney-21 cells. The cells were incubated and observed daily for detecting cytopathic effects (CPEs). The virus was harvested after detecting CPEs. Forty baby Swiss mice (age, 1-2 weeks) were divided into three completely separated groups under pathogen-free conditions and biosafety level 3. The first group of 15 mice was inoculated intraperitoneal (I/P) with 100-mL putative harvested virus suspension. The second positive control group of 15 mice was inoculated with a suspension of known isolated serotype O FMDV, and the third negative control group of 10 mice was inoculated with 100-mL PBS I/P. All groups were observed thrice daily for clinical symptoms and mortality. Brain samples were collected during postmortem examination. Parts of the collected tissues were fixed for histopathological examination, and another part was homogenized for viral isolation and PCR.

## Results

### Clinical findings

The important point to remember is that all FMDV-infected calves in this study were healthy before the FMDV outbreaks based on their history, appetite, gait, defecation, and urination. An overview of the clinical findings of the field outbreak investigations suggests dysautonomia. Signs of dysautonomia were recorded in malignant cases with or without the classic symptoms of FMDV infection, whereas cases that recovered exhibited classic signs of FMDV infection without any evidence of dysautonomia (Table-3).

**Table-3:** Observed clinical alterations in malignant FMDV cases and FMDV recovered calves.

Clinical findings		Malignant cases with dysautonomia (134)				Recovered cases calves (201)	

		Acute (54)	Sub-acute (80)				
		
		No.	%	No.	%	No.	%
State of appetite	In appetence	0 of 54	0	60 of 80	75	172 of 201	85.57
	Anorexia	54 of 54	100	20 of 80	25	29 of 201	14.43
Sunken eye	Mild	5 of 54	9.25	66 of 80	82.5	30 of 201	14.92
	Moderate	18 of 54	33.33	8 of 80	10	5 of 201	2.49
	Severe	32 of 54	59.25	6 of 80	7.5	0 of 201	0
Gait	Incoordination and bradykinesia	50 of 54	92.59	46 of 80	57.5	0 of 201	0
Posture	Sternal recumbency with extended neck	39 of 54	72.22	22 of 80	27.5	0 of 201	0
Examination of mouth	Lesions	3 of 54	5.55	65 of 80	81.25	201 of 201	100
	Salivation	0 of 54	0	65 of 80	81.25	201 of 201	100
Examination of foot	Lesions	0 of 54	0	49 of 80	61.25	201 of 201	100
	Lameness	0 of 54	0	47 of 80	58.75	201 of 201	100
Wide fluctuation in body temperature within a day		54 of 54	100	80 of 80	100	0 of 201	0
Shallow loud breathing and tachypnea		54 of 54	100	80 of 80	100	65 of 201	32.33
Cardiac arrhythmias (Tachy and brady arrhythmias)		54 of 54	100	80 of 80	100	0 of 201	0
Abnormal heart sounds		0 of 54	0	0 of 80	0	0 of 201	0
Heat intolerance		0 of 54	0	7 of 80	8.75	0 of 201	0
Hirsutism (shaggy coat with longer hair)		0 of 54	0	13 of 80	16.25	0 of 201	0
Muscle fasciculations		30 of 54	55.55	5 of 80	6.25	0 of 201	0
Urination	Oliguria	30 of 54	55.55	53 of 80	66.25	22 of 201	10.95
	Anuria	24 of 54	44.44	27 of 80	33.75	0 of 201	0
Anal tone	Reduced	6of 54	11.11	69 of 80	86.25	0 of 201	0
	Absent	48 of 54	88.88	0 of 80	0	0 of 201	0
Feces	Hard dark feces	49 of 54	90.74	67 of 80	83.75	0 of 201	0
Fate	Excessive periodic bellowing before death	38 of 54	70.37	72 of 80	90	Recovery	
	Found dead	16 of 54	29.62	8 of 80	10		

FMDV=Foot-and-mouth disease virus

The clinical findings of dysautonomia in the malignant cases varied widely between animals, and within days or even hours, cases of acute and subacute dysautonomia were observed. Acute forms (several hours to 1 day) were characterized by the absence of oral and foot lesions, sternal recumbency, sometimes with extended head and neck on the ground, and severe incoordination and bradykinesia if the animal was forced to walk. Loud and rapid breathing sounds that could be heard from a distance, unexplained dehydration, fluctuating body temperatures that largely reflected the environmental temperature, mostly subnormal temperature with cold extremities, and muscle fasciculation before death were observed. Cardiac arrhythmias with variable periods of regularity were also observed, along with complete gut and sphincter dysfunction, manifested as constipation and loss of anal tone.

In some cases, the anus was wide open as if being opened by a speculum, with the appearance of rectal mucosa free from feces and mucus for ~10 cm from the anus, after which fecal mass was impacted. Anuria and oliguria were also observed. Finally, lateral recumbency, excessive periodic bellowing, and death occurred.

In cases of subacute dysautonomia, the clinical course lasted for 2-14 days, and though several symptoms may not have been observed, death or slaughter was the only fate. Most cases of subacute dysautonomia started with the classic symptoms of FMDV infection: Inappetence to anorexia, fever, oral and foot lesions, salivation, and lameness; however, when dysautonomia occurred, salivation and fever usually subsided, and fluctuating body temperatures were observed. Cardiac arrhythmias and tachypnea were detected during the clinical course. Heat intolerance syndrome (HIS) and hirsutism were observed in cases where the clinical course lasted for more than 10 days. Oliguria and loss of anal tone with infrequent dry, firm, and dark feces sometimes hanging from the anus were frequently detected. Note that some cases of subacute dysautonomia expressed moderate cardiopulmonary disease (i.e. moderate tachypnea, tachycardia, and cardiac arrhythmia), anuria or oliguria, and constipation with normal anal tone, causing some cases to be missed until death. Unexpectedly, some animals with subacute dysautonomia still stood, ate, and ruminated until death.

Auscultation of the lungs in malignant cases of FMDV infection revealed either exaggerated vesicular sounds or abnormal bronchial respiratory sounds. Cardiac arrhythmias were observed during heart auscultation. During an attempt to warm the calves with subnormal temperatures and cold extremities, the calves began to pant vigorously and were suffering from HIS, with rapidly elevating body temperatures.

### Postmortem examination

**Figure-1:**
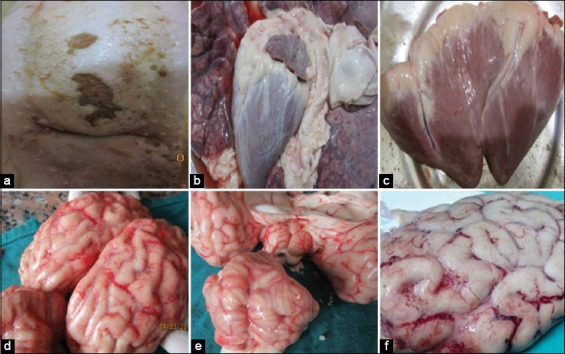
Gross findings in malignant cases; ulceration in the tongue (a), congestion of lung (b), heart with white streaks on myocardium (c), and the brain showing variable degrees of congested blood vessels of meninges (d, e, and f).

Gross findings in malignant cases revealed ulcerations on the tongue and dental pad (Figure-1a). The lungs revealed cranioventral symmetrical congestion on both sides, which extended dorsally in some cases (Figure-1b). The hearts were grossly normal with or without white streaks on the myocardium (Figures-1b and c). The brain revealed a variable degree of meningitis with congested blood vessels and no evidence of inflammation (Figures-1d-f.

### Histopathological examination

**Figure-2:**
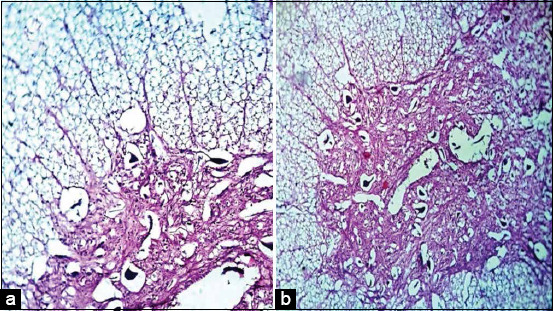
Histopathological features of spinal cord of foot and mouth disease malignant cases; white matter showing demyelination of most nerve fibers HE 1200× (a) and gray matter showing perivascular and perineuronal edema plus neuronal degeneration HE 300× (b).

**Figure-3:**
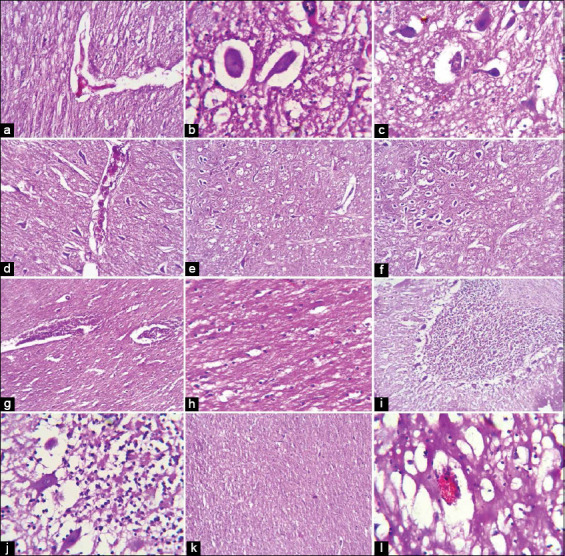
Histopathological features of the brain of foot and mouth disease malignant cases; Pons showing congestion (arrow) and demyelination (arrowhead) HE 300× (a), neuronal degeneration (arrow) with perineural edema (arrowhead) HE 1200× (b), medulla oblongata showing shrunken neurons (arrow) and perineural edema (arrowhead) with mild gliosis HE 1200× (c), severe congestion (arrow) with perineural edema (arrowhead) HE 300× (d), axonal demyelination and vacuolations HE 300× (e), perineural edema (arrow) and encephalomalacia (arrowhead) HE 300× (f), hypothalamus, showing congested blood vessels (arrow) with axonal demyelination (arrowhead) HE 300× (g), the proliferation of glial cells (arrow) HE 1200× (h), cerebellum showing necrotic Purkinje cells (arrow) (HE 300× I and HE 1200× J), cerebral cortex showing gliosis (arrow) HE 300× (k), congestion (arrow) with encephalomalacia, and demyelination (arrowhead) HE 1200× (l).

**Figure-4:**
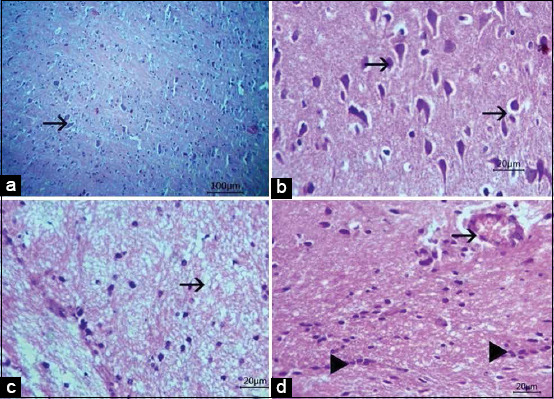
Histopathological features of the mice brain inoculated with putative isolated foot and mouth disease virus from suspected samples in Koch’s postulates. The cerebral cortex showing normal histologic brain, bar 100 μm (a), neuronal degeneration (arrow), bar 20 μm (b), demyelination and encephalomalacia (arrow) bar 20 μm (c), congestion (arrow) and proliferation of glial cells, bar 20 μm (arrowhead) (d).

Lung and heart tissues revealed marked pulmonary edema and lymphocytic myocarditis with cardiac muscle fiber degeneration, respectively. Spinal cord white matter revealed demyelination of most nerve fibers, whereas the gray matter revealed perivascular and perineuronal edema and neuronal degeneration (Figures-2a and b). Brain tissue revealed congestion and various degenerative changes with limited or no inflammatory response. The pons and medulla oblongata, hypothalamus, cerebellum, and cerebrum revealed demyelination, neuronal degeneration, shrunken neurons, perineural edema, gliosis, necrotic Purkinje cells, vacuolations, and encephalomalacia (Figures-3a-l). In contrast to negative control baby mice (Figure-4a), histopathological examination of FMDV-inoculated baby mice confirmed neurological damage, revealing neurodegeneration, demyelination, and encephalomalacia of the nervous tissue (Figures-4b-d).

### Molecular examination

**Figure-5:**
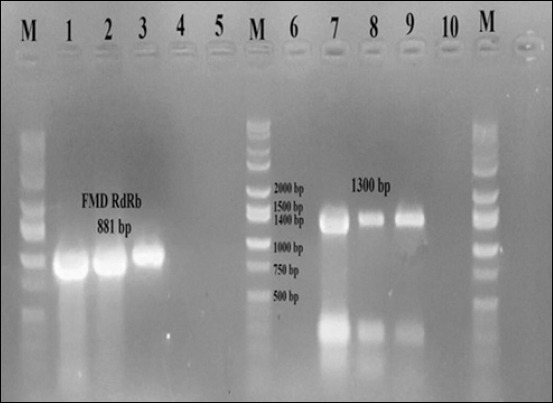
Representative polymerase chain reaction bands of foot and mouth disease RdRb (3D) gene and 1D gene, (1 and 7) brain samples, (2 and 8) spinal cord samples, (3 and 9) positive control, (5 and 10) negative control.

Of the 372 examined RNA samples obtained from suspected animal saliva and mouth swabs, 335 were positive for 3D primers. Further examination using a 1D primer revealed the absence of SAT2 and A serotypes. All 335 positive samples revealed O serotype in all noted outbreaks (Figure-5). The examined RNA samples obtained from dead or slaughtered animals were positive for the 1D primer of O serotype. PCR was positive in all heart and nervous tissue samples, and 60% of lung tissues were positive. No bacterial or BHV4 infections were detected in any of the PCR examined samples.

### Koch’s postulates

All mice in the first and second groups died within 2-7 days after displaying clinical signs of FMDV infection: Humped posture, stunted growth, neurological signs, tremors, ataxia, and paralysis of the hind limbs. Re-isolation of FMDV from brain tissues of both groups succeeded, and CPEs were observed on the 3^rd^ day of incubation, in addition to the previously mentioned histopathological alterations in the brains of the baby mice.

## Discussion

In this study, clinical findings of dysautonomia were meticulously observed in malignant cases of FMDV infection. Dysautonomia has not been reported in calves, and in our opinion, it should be considered a warning sign in malignant cases of FMDV infection. This study was based on an unorthodox theory, and further studies are necessary to conclude the causal association between neurodegeneration and FMDV infection in malignant cases. Here, the available laboratory trials were conducted to understand the possible cause/s of dysautonomia, whether viral, bacterial, or as a result of concurrent infections.

Cases of acute and subacute dysautonomia due to different causes were previously reported in several species, including equines [17,18], cats [19], dogs [20], sheep [21], and alpacas [22]. The symptoms of dysautonomia are usually “invisible” or “enigmatic” to the untrained eye, to the owners, and sometimes even to veterinarians and researchers. Special interest was given to the signs that indicated dysautonomia, such as tachypnea, cardiac arrhythmias, reduced or absent anal tone, and oliguria or anuria. Sluggish intestinal motility with reduced bowel movement, difficulty in defecation with excessive straining, and incomplete emptying may be due to gastrointestinal motor dysfunction [23]. Furthermore, in humans, anuria due to bladder dysfunction, anorectal dysfunction, and loss of anal tone has been attributed to brain pathologies [24,25]. The tachypnea and cardiac arrhythmias that animals presented before death were direct results of alterations in autonomic function [26]; moreover, with the use of cardiac biomarkers in FMDV-infected calves, myocardial damage may occur regardless of whether the calves die of infection [27]. Cardiac arrhythmias recorded in this study can be described as cardiogenic dysautonomia. The ANS plays an important role in controlling heart rate variability, which is an important risk factor for arrhythmias, myocardial infarction, heart failure, and sudden cardiac death [28].

In support of these theories, a study has reported that stimulation of the anterior hypothalamus in cats causes a burst of parasympathetic activity, whereas in cases of bradycardia, stimulation of the lateral hypothalamus increases heart rate, and causes electrocardiogram alterations [29]. The noted locomotor disorders, lethargy, bradykinesia during walking, and recumbency are all common signs of dysautonomia in humans [30]. Locomotor disorders were previously detected during serotype O FMDV outbreaks without definite explanations for why sick animals exhibited these disorders, and dead gazelles were concentrated in and around water sources and under vegetation [31], indicating heat intolerance. Likewise, early studies have reported that cattle with HIS following an FMD attack lose the condition and show a great tendency to stand in ponds or water troughs [32], indicating their inability to tolerate environmental temperatures that unaffected animals can tolerate.

The HIS recorded here was similar to previously recorded cases of chronic sequelae of FMD in cattle. Cattle with HIS cannot recover, and the pathogenesis of this syndrome is unknown, though impaired thermoregulation was suspected by some researchers [32]. Previously, HIS was attributed to the effects of the FMDV on the hypothalamic–pituitary–endocrine axis that may also cause permanent damage and metabolic derangement [33]. Hirsutism is another syndrome that can accompany HIS and was observed in this study, especially when the clinical course lasted for 10-14 days.

Meningitis recorded in this study may be similar to an earlier study that has recorded some degree of petechial hemorrhage in meninges in the postmortem examination of cattle with HIS [32]. Severe dehydration was reported in some gazelles, even though they were found adjacent to water. These phenomena could be attributed to the animals’ inability to drink due to lingual muscular changes [31].

Histopathological findings in the lungs revealed pulmonary congestion and edema, similar to the findings of recent studies [34]. Neurogenic pulmonary edema has been explained by an increase in pulmonary vascular pressure due to sympathetic stimulation, with a subsequent increase in pulmonary endothelial permeability [35,36]. Myocarditis may occur due to direct invasion of the myocardium by a cardiotropic virus, causing local inflammation [37]. Concerning virus isolation, the variations in the amount of viral RNA detected in different tissues can be attributed to the kinetics of FMDV infection and elimination in bovine tissues reflected by the number of days after infection [38]. The percentage varied as animals did not die simultaneously after infection. The heart and brain tissue of malignant cases still had the virus, which indicates the tropism of the virus in these tissues.

Many fundamental questions remain to be answered: Is the virus originally neurotropic? How does the virus invade the CNS? Can neurodegeneration be attributed to viral damage of neurons or to an immune attack on virally infected cells? As these questions cannot be answered confidently, these mechanisms must be fully understood. Isolation of FMDV from the brains of calves was supported by a previous study that has reported the presence of FMDV in the pituitary gland and CNS of experimentally infected cattle [39]. *Picornaviruses* can enter the CNS by retrograde axonal transport when *Picornaviruses* enter the neuromuscular junction or directly from the blood [40]. Unfortunately, the conclusive proof for neurotropism of FMDV lies in the localization of the FMDV antigen in nervous tissue by immunohistochemistry, and no facilities exist for this technique.

The FMDV was confirmed using PCR and Koch’s postulate procedures using unweaned baby mice. Mice were highly susceptible to FMDV infection and acted as a perfect model in Koch’s postulate procedure when inoculated intraperitoneally. The FMDV could cause neurological signs and histopathological lesions in the brain of inoculated mice, supporting the ability of the FMDV to cause neurological damage. The FMDV has a highly destructive effect on cardiac and skeletal muscles of young mice. Damage caused necrosis in neuromuscular nerve endings of muscle fibers, leading to degeneration, clumping, and fragmentation of neurofibrillary terminations or the absence of nerve endings in severely damaged areas [41]. Previously, viral adsorption was tested in minced and homogenized mouse kidney, lung, muscle, and brain tissues. The adsorption of the FMDV was proven with a higher percentage in minced brain and muscle tissues than in kidney and lung tissues. A greater amount of cell-associated virus was recovered from the brain and muscle tissues than in the kidney and lung tissues [42].

Differential molecular diagnoses of BHV4 and bacterial infections were performed and excluded using PCR. The probability of BHV4 infection decreased after clinical examination because of the absence of characteristic signs [43]. Putative nervous dysfunction was caused by FMDV infection, mostly related to abnormalities in the ANS with consequent constipation, anuria, cardiac arrhythmias, fluctuating temperature, and the presence or absence of classic signs; moreover, the symptoms and deaths were associated with the period of FMDV outbreaks only, and the recorded neurological signs in mice inoculated with the FMDV were similar to those reported in mice inoculated intraperitoneally with another neurotropic *picornavirus* [44].

In our opinion, the absence of clear nervous manifestations (in the form of excitation signs) and the presence of signs of cardiopulmonary dysfunction are possible reasons behind why CNS involvement has not been considered in the past; however, the poorly understood syndrome HIS — also referred to as “hairy panter” syndrome — that was reported in some cases of cattle who recovered from FMDV infection suggests that the brain is badly affected. The FMDV has multiple tissue tropisms, including the epithelium and muscle (e.g. myotropism, myocardium, diaphragm, and skeletal muscle). Myotropism may occur with or without concurrent vesicular lesions. The absence of vesicles indicates tropism (muscle, but not epithelium), and this study suggests a new tropism to FMDV (neurotropism), also occurring in the absence of vesicular lesions.

Unlike DNA viruses, RNA viruses are the masters of mutation. RNA polymerases cannot correct any errors that arise, allowing for the rapid rate of mutation [45]. In recent outbreaks in Egypt, high morbidity and mortality rates have occurred in cattle of different ages, indicating higher virulence [46].

Further investigations are required to determine whether neurodegeneration is attributable to viral damage of neurons or to immune attack on virally infected cells. In any case, the struggle between viruses and brain cells may be viewed as a battle, with the survival of either of the brain cells or viruses dependent on complex strategies. Host genetic factors may influence the type and quality of immune response and may explain why only a small percentage of infected cases develop neurological disease in certain diseases [9].

## Conclusion

To the best of our knowledge, this is the first study that has observed the relationship between FMDV infection and neurodegenerative changes in calves. This study reported the clinical alterations associated with dysautonomia in calves. Further studies are required to conclude the causal association between neurodegeneration and dysautonomia in FMDV-infected calves.

## Authors’ Contributions

YB: Hypothesis generation, YB, NS, and WE: Examined diseased calves, samples collection and executed most of the laboratory work. NE: Performed the histopathological examination. YB, AM, and AA: Drafted the manuscript and analyzed the data. All authors drafted, read, and approved the final manuscript.
